# Coxsackievirus B3-Induced m^6^A Modification of RNA Enhances Viral Replication via Suppression of YTHDF-Mediated Stress Granule Formation

**DOI:** 10.3390/microorganisms12112152

**Published:** 2024-10-26

**Authors:** Guangze Zhao, Huifang M. Zhang, Yankuan T. Chen, Kerry Shi, Sana Aghakeshmiri, Fione Yip, Honglin Luo, Bruce McManus, Decheng Yang

**Affiliations:** 1 Department of Pathology and Laboratory Medicine, University of British Columbia, Vancouver, BC V6T 1Z4, Canada; guangze.zhao@hli.ubc.ca (G.Z.); mary.zhang@hli.ubc.ca (H.M.Z.);; 2 Centre for Heart Lung Innovation, University of British Columbia, St. Paul’s Hospital, 1081 Burrard Street, Vancouver, BC V6Z 1Y6, Canada

**Keywords:** coxsackievirus B3, N6-methyladenosine, HuR, 3D, reader protein YTHDFs, stress granule, dsRNA

## Abstract

N6-methyladenosine (m^6^A) is the most prevalent internal RNA modification. Here, we demonstrate that coxsackievirus B3 (CVB3), a common causative agent of viral myocarditis, induces m^6^A modification primarily at the stop codon and 3′ untranslated regions of its genome. As a positive-sense single-stranded RNA virus, CVB3 replicates exclusively in the cytoplasm through a cap-independent translation initiation mechanism. Our study shows that CVB3 modulates the expression and nucleo-cytoplasmic transport of the m^6^A machinery components—METTL3, ALKBH5 and YTHDFs—resulting in increased m^6^A modifications that enhance viral replication. Mechanistically, this enhancement is mediated through YTHDF-driven stress granule (SG) formation. We observed that YTHDF proteins co-localize with human antigen R (HuR), a protein facilitating cap-independent translation, in SGs during early infection. Later in infection, YTHDFs are cleaved, suppressing SG formation. Notably, for the first time, we identified that during early infection CVB3’s RNA-dependent RNA polymerase (3D) and double-stranded RNA (dsRNA) are stored in SGs, co-localizing with HuR. This early-stage sequestration likely protects viral components for use in late-phase replication, when SGs are disrupted due to YTHDF cleavage. In summary, our findings reveal that CVB3-induced m^6^A modifications enhance viral replication by regulating YTHDF-mediated SG dynamics. This study provides a potential therapeutic strategy for CVB3-induced myocarditis.

## 1. Introduction

Methylation at the N^6^ position of adenosine (m^6^A) is the most abundant internal mRNA modification, which is formed during various cellular stress conditions including viral infection. This modification process is dynamic and reversible, and evolutionarily conserved across different organisms [1,2,3]. The level of this methylation is regulated by its modification machinery, including methyltransferases (writers) and demethylases (erasers). After modification, the m^6^A sites are recognized by the m^6^A-binding proteins YTHDF1-3, YTHDC1 and YTHGC2 (readers) through its YTH domain [4,5,6,7]. These reader proteins play critical roles in a variety of biological processes, such as mRNA splicing [5], RNA stability [8,9,10], structure switch [11], nuclear export [12] and translation control [13,14]. Recently, it has also been reported that reader proteins YTHDF1-3 can promote the formation of cytosol stress granules (SG), a cellular response to stress conditions such as heat shock, hypoxia, irradiation, toxin, infection, etc. [15,16,17].

SGs are nonmembranous granular aggregates formed in the cytoplasm of eukaryotic cells when exposed to a variety of environmental stress conditions. They are the storage place for mRNA-protein complexes consisting of m^6^A-modified mRNA and proteins/enzymes involved in translation [18]. The stalled mRNAs and proteins may be reactivated when the stress condition is removed. Therefore, SG formation is a cellular strategy to protect its translation machinery for better survival [19,20]. In the case of viral infection, SG components contain many signal molecules involved in type I interferon signal pathways, such as RIG-1, MDA5, LGP2 and PKR [21,22]; thus, SG is recognized as the platform for antiviral innate immune response [19,23,24]. m^6^A modification is a novel layer of gene regulation in various organisms including viruses, particularly nuclear viruses such as simian virus 40 (SV40), influenza virus and HIV [25,26,27]. However, the effect of m^6^A modification on Coxsackievirus B3 (CVB3), a cytoplasmic virus, is unclear.

CVB3 is a member of the enterovirus genus in the *Picornaviridae* family. It infects multiple organs and causes different diseases, such as myocarditis, meningitis and pancreatitis. However, the most common and deadly disease is myocarditis, particularly in children and adolescents. This viral genome is a positive single-stranded RNA molecule, and its transcription is regulated by viral RNA-dependent RNA polymerase 3D. CVB3 RNA translation is initiated by a cap-independent, IRES (internal ribosome entry site)-dependent mechanism [28], requiring IRES trans-activating factors (ITAF) [29,30]. Further, it is well documented that ITAFs are key factors impacting cellular stress response [31]. These factors, such as human antigen R (HuR), poly(rC)-binding protein (PCBP) and polypyrimidine tract-binding protein (PTB), are stored in SGs or the processing body during cellular stress conditions [32,33,34]. Our previous studies and others found that CVB3 infection causes endoplasmic reticulum stress, induces m^6^A modification of RNA and benefits viral replication [35,36,37]. In addition, enterovirus infection induces SG formation in the early phase of infection but suppresses SG production at later time points [22,38,39]. These studies conducted previously have raised several fundamental questions as follows: what is the overall effect of m^6^A modification and SG production on CVB3 replication? What is the underlying mechanism of phase-dependent action of SG formation on controlling CVB3 replication? What is the linkage between m^6^A modification and SG formation in the modulation of viral replication?

In this study, we first verified the significant induction of m^6^A modification of RNA in CVB3-infected HeLa cells, HL-1 cardiomyocytes and mouse hearts. Through methylated RNA immunoprecipitation (MeRIP)-RT-qPCR using seven pairs of virus-specific primers covering the entire CVB3 RNA genome, we further confirmed that CVB3-induced viral RNA m^6^A modifications are mainly located at the 3′ untranslated region (3′UTR), stop codon region and the 5′UTR. We further demonstrated that methyltransferases and demethylases are differentially regulated by CVB3 infection and are responsible for the upregulation of m^6^A modification rates on CVB3 RNA. This conclusion was solidified by experiments on overexpressing or silencing methyltransferase and demethylase. Further investigations found that these modifications have a positive effect on the CVB3 life cycle. This conclusion is evidenced by the resultant increases in CVB3 2A transcription, VP1 protein synthesis and infectious viral particle formation. In further revealing the mechanism underlying the enhanced CVB3 replication, we found that the suppression of antiviral SG formation at a later phase plays a pivotal role. Specifically, we revealed that this SG suppression is linked to the cleavage of the m^6^A reader protein during CVB3 infection since silencing and overexpression of m^6^A reader protein YTHDF2 could enhance and inhibit SG formation, respectively. By immunofluorescent staining, we found for the first time that 3D and CVB3 double-stranded RNA (dsRNA, is a replication intermediate during CVB3 replication.), are stored in SGs and colocalized with HuR, an ITAF protein, at early stages of infection. This early-stage sequestration likely protects viral components for use in late-phase replication when SGs are disrupted due to YTHDF cleavage. In summary, our findings reveal that CVB3-induced m^6^A modifications enhance viral replication by regulating YTHDF-mediated SG dynamics.

## 2. Materials and Methods

### 2.1. Virus, Cells and Mice

CVB3 (Kandolf strain) was routinely propagated in HeLa cells (ATCC). The virus supernatant was prepared by three freeze–thaw cycles of the infected cells, centrifuged to remove cell debris, and stored at −80 °C. Virus titers were determined by plaque assay prior to infection as previously described [35]. For in vivo studies, all mouse experiments were performed according to the animal experimental protocols approved by the Animal Care Committee of Faculty of Medicine, University of British Columbia (protocol number: A16-0093). Male A/J mice (4 weeks old) were purchased from the Jackson Laboratory (Bar Harbor, ME, USA) and infected via intraperitoneal injection with CVB3 at 10^5^ pfu (plaque forming unit) or sham-infected with saline. HeLa cells were grown in Dulbecco’s Modified Eagle’s Medium (DMEM) supplemented with 100 μg/mL penicillin, 100 μg/mL streptomycin, 2 mM glutamine, and 10% fetal bovine serum (FBS) (Life Technology, Carlsbad, CA, USA). The cells were infected with CVB3 at 10 MOI (multiplicity of infection) and collected at different time points. The HL-1 cell line, a cardiac muscle cell line established from a mouse atrial cardiomyocyte tumor lineage, was a gift from Dr. William C. Claycomb (Louisiana State University Health Science Center, New Orleans, LA, USA). The cells were maintained in Claycomb medium supplemented with 10% FBS (JRH Biosciences, Lenexa, KS, USA), 100 μg of penicillin/streptomycin/mL, 0.1 mM norepinephrine (Sigma, Burlington, VT, USA), and 2 mM L-glutamine (Invitrogen, Waltham, MA, USA). The HL-1 cells were infected with CVB3 at an MOI of 50.

### 2.2. siRNA and Plasmid Transfections

The siRNAs targeting human METTL3 (sc-92172), YTHDF2 (sc-78661) or ALKBH5 (sc-93856) were purchased from Santa Cruz Biotechnology (Dallas, TX, USA). The plasmids used in this study include pcDNA3-flag-METTL3 (Addgene, #53739), pcDNA3/Flag-METTL14 (Addgene, #53740), p-FRT/TO/Flag/HA-YTHDF2 (Addgene, #38089), and pcDNA-ALKBH5 (a gift from Dr. Ge Shan, School of Life Sciences, University of Science and Technology of China, Hefei, China). siRNA transfections were performed under optimal conditions following the manufacturer’s instructions. Next, 2 × 10^5^ cells were grown in 6-well plates at 37 °C overnight. Once 30–40% confluency was reached, the cells were washed with PBS, and overlaid with transfection complex containing OPTI, siRNA, and Lipofectamine (Life Technology) for 36 h. The transfection medium was then replaced with DMEM containing 10% FBS and incubated for 36 h prior to collection. Plasmid transfections were conducted using the same protocol, with Lipofectamine 2000 (Invitrogen) as the transfection reagent.

### 2.3. RNA Isolation and Measurement of N6-Methyladenosine

Cellular RNA containing CVB3 RNA was extracted from both cultured cells and mouse heart tissues. For cultured cells, HeLa cells were collected 5 h post infection (hpi), and HL1 cells were collected 24 hpi. RNA extraction from these cells was performed using the TRIzol reagent (Invitrogen, Waltham, MA, USA), adhering to the manufacturer’s protocol. For mouse heart tissues, samples were obtained 7 days pi. RNA was isolated from the ventricle tissues (the area most susceptible to CVB3 infection) using the RNeasy Mini Kit (Qiagen, Metin Colpan, Schumacher), following the manufacturer’s guidelines. m^6^A levels were measured using the EpiQuik m^6^A RNA Methylation Quantification Kit (EpiGentek, Farmingdale, NY, USA) in accordance with the manufacturer’s instructions.

### 2.4. Real-Time qPCR

Total RNA was extracted from cultured cells using the Monarch Total RNA Miniprep Kit (NEB, Ipswich, MA, USA), following the manufacturer’s protocol. Reverse transcription of 5 μg of RNA was performed to synthesize cDNA using the All-in-One cDNA Synthesis SuperMix (Bimake, Houston, TX, USA). RT-qPCR was conducted with 2× SYBR Green qPCR Master Mix (Low Rox), utilizing 100 ng of cDNA and 1 μM of each primer for amplification. The expression of the target genes was quantified, and GAPDH served as the reference gene to standardize the results.

### 2.5. Western Blot Analysis

Western blotting was conducted using standard protocols as previously described [35]. Cultured cells growing in 6-well plates were briefly washed with cold PBS before the addition of lysis buffer (0.025 M Tris-HCl, pH 8.0, 137 mM NaCl, 10% glycerol, 1 mM EDTA, 1 mM EGTA, 1% Triton X-100, and proteinase inhibitor cocktail). After incubation of the cells in lysis buffer on ice for 20 min, the supernatant containing the proteins was collected by centrifugation at 14,000× *g* for 15 min at 4 °C. Protein concentrations were determined by Bradford assay (Bio-Rad, Hercules, CA, USA). For mouse hearts, the heart tissue sections were first rinsed with PBS to remove the blood and then lysed in RIPA lysis buffer using TissueLyser LT (Qiagen). Samples were briefly sonicated at 40 Hz for 30 s to release the proteins. The protein-containing supernatants were collected by centrifugation and protein concentrations were determined as described above. Equal amounts of protein were separated by 8–12% SDS-polyacrylamide gel electrophoresis (SDS-PAGE) and transferred onto nitrocellulose membranes (BioTrace NT nitrocellulose, Pall Corporation, New York, NY, USA). The membranes were blocked with 5% skim milk in PBS and incubated overnight with shaking using one of the following primary antibodies: monoclonal mouse anti-β-actin (Sigma-Aldrich, St. Louis, MO, USA), monoclonal mouse anti-VP1 (M47, Reutlingen, Germany). Polyclonal rabbit anti-human YTHDF1 (66745-1AP), YTHDF2 (24744-1AP), YTHDF3 (25537-1-AP), METTL3 (15073-AP) and METTL14 (23158-1-AP) (Proteintech, Rosemont, IL, USA); polyclonal rabbit anti-human ALKBH5 (Abcam, Cambridge, United Kingdom); monoclonal mouse anti-Flag tag (Sigma) and monoclonal mouse anti-3D antibody (GeneTex, Irvine, CA, USA). After three washes, each membrane was incubated with an appropriate secondary antibody (goat anti-mouse or goat anti-rabbit IgG) conjugated to horseradish peroxidase (Santa Cruz). Signals were detected using the ECL method according to the manufacturer’s instructions (BIO-RAD, Clarity^TM^ Western ECL Substrate).

### 2.6. Immunofluorescence Staining and Confocal Microscopy

HeLa cells were proliferated on μ-Slide 8-well coverslip (ibidi. Cat. No. 80826). The cells were infected with CVB3 for 3 h before staining when they were at approximately 70% confluency. The cells were fixed with 4% paraformaldehyde and permeabilized in methanol/acetone (1:1) at −20 °C for 20 min, followed by two washes with PBS and then blocked with bovine serum albumin (Sigma) in PBS for 1 h at room temperature. To detect the co-localization of YTHDF2 and HuR in SG, the cells were incubated with specific primary antibodies, YTHDF2 and HuR, at 4 °C overnight. Afterwards, the cells were washed with PBS five times at room temperature and stained with a goat anti-rabbit IgG labeled with Alexa Fluor 488 (green) or goat anti-mouse IgG with Alexa Fluor 594 (red) (Invitrogen). Nuclei were stained with 4′,6′-diamidine-2′-phenylindole dihydrochloride (DAPI) (blue) (Vector Laboratories, Newark, CA, USA). Cells were observed with a Leica SP2 AOBS confocal microscope as described previously [40].

### 2.7. Immunoprecipitation

HeLa cells were infected with CVB3 at an MOI of 10 for 3 h. The cell lysates were obtained using lysis buffer (25 mM Tris HCl, 150 mM NaCl, 1 mM EDTA, 1% NP40, 5% glycerol, pH 7.4) (Thermo scientific, Burnaby, Canada), with protease inhibitor cocktail (Roche, 04693132001). The lysates were centrifuged at 14,000× *g* for 20 min at 4 °C. After preclearing for 2 h, the supernatants were immunoprecipitated overnight by rotating with agarose beads coated with the anti-YTHDF2 antibody (24744-1AP, Proteintech, Rosemont, IL, USA). The immunocomplexes captured on the beads were processed to detect the interaction between YTHDF2 and HuR following the manufacturer’s protocol (Thermo scientific co-IP kit, cat log # 8828). Briefly, the beads were washed, eluted, and neutralized with respective buffers provided in the kit. Finally, proteins bound to the beads were boiled in SDS sample buffer and subjected to SDS-PAGE analysis using anti-HuR antibody (Invitrogen, catlog#39-0600) or anti-YTHDF2. The interaction between YTHDF2 and HuR was further confirmed by immunoprecipitation using the anti-HuR antibody to pull down the immunocomplexes and then identified by Western blot using the anti-YTHDF2 antibody following the same protocols.

### 2.8. Viral Plaque Assay

Viral titers were determined by plaque assay as described previously [41]. HeLa cells were seeded onto 6-well plates (8 × 10^5^ cells/well) and incubated at 37 °C for 20 h. Approximately 80–90% confluent cells were washed with PBS twice and then overlaid with 500 μL of serially diluted viral supernatant. The cells were incubated at 37 °C for 60 min and the supernatants were removed. The cells were washed with PBS twice and then overlaid with 2 mL of sterilized soft Bacto-agar-minimal essential medium. After the agar was solidified, the cells were cultured at 37 °C for 72 h with bottom of the culture plate up. After this incubation, Bacto-agar was removed and the cells were fixed with Carnoy’s fixative solution for 30 min, and stained with 1% crystal violet for 2 min. Finally, the cells were washed with PBS, the plaques were counted and the amount of viral plaque forming units (pfu) per ml was calculated.

### 2.9. Statistical Analysis

Statistical analysis was carried out using Prism 10 (GraphPad Software, San Diego, CA, USA). For comparisons across multiple groups, one-way ANOVA was applied, followed by Tukey’s post hoc test to evaluate specific group differences. To analyze differences between unpaired groups, Student’s *t*-test with Welch’s correction was utilized. The data are presented as mean values ± standard error of the mean (SEM) from five technical replicates, with biological replicates set at n = 3. Statistical significance was defined as *p* < 0.05 (indicated by *), with ** *p* < 0.01, *** *p* < 0.001, and **** *p* < 0.0001.

## 3. Results

### 3.1. CVB3 Infection Induces m^6^A Modifications of Cellular RNAs in HeLa Cells, HL-1 Cardiomyocytes and Mouse Hearts

**Figure 1:**
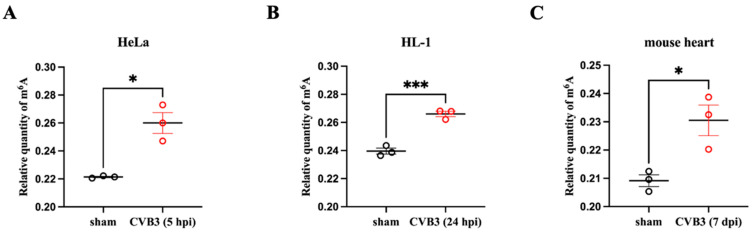
Relative levels of N6-methyladenosine (m^6^A) modification increase after CVB3 infection. Total RNAs were isolated from CVB3-infected or sham-infected HeLa cells (**A**), mouse HL-1 cardiomyocytes (**B**), and mouse hearts (**C**) at the indicated time points post infection (pi). The relative m^6^A level of each sample was determined using the EpiQuick m^6^A quantification kit. Data are presented as means ± SEM, n = 3, * *p* < 0.05, *** *p* < 0.001.

To investigate whether CVB3 RNA is modified by m^6^A during viral infection, we first detected whether CVB3 infection could increase m^6^A modification levels in total RNAs of CVB3-infected cells. To gain a broader insight into cell-type specificity of m^6^A modification, we measured the relative levels of m^6^A following CVB3 infection in two cell lines (human HeLa cells and mouse HL-1 cardiomyocytes) and a mouse model. Total cellular RNAs containing CVB3 RNA were extracted from HeLa cells infected with CVB3 at 10 MOI for 5 h and HL-1 cells infected with CVB3 at 50 MOI for 24 h. The RNAs from the sham-infected sample were also prepared in parallel as a control. For the animal model, A/J mice were infected with CVB3 at 10^5^ pfu or sham-infected with saline for 7 days. Mouse hearts were harvested for total cellular RNA isolation. As shown in Figure 1, CVB3-infected HeLa cells (Figure 1A), HL-1 cells (Figure 1B) and mouse hearts (Figure 1C) all showed significantly increased m^6^A levels compared to the controls. These data suggest that CVB3 infection induces RNA m^6^A modification.

### 3.2. m^6^A Modification of CVB3 RNA Is Enriched at Its Stop Codon and 3′UTR Regions During the Course of Infection

**Figure 2:**
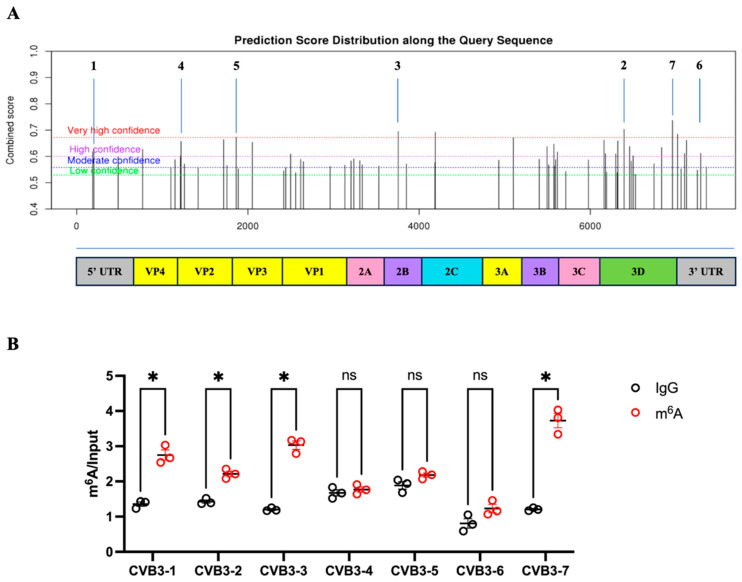
Mapping of m^6^A sites on CVB3 genomic RNA. (**A**) m^6^A modification sites were predicted using an online mammalian m^6^A prediction tool (SRAMP). The prediction is based on the DRACH motif (where D = A, G, or U; R = G or A; H = A, C, or U) and additional sequence as well as structural features within the CVB3 sequence. The predicted m^6^A sites labeled as “Very high confidence” have a 1% false positive rate, making them highly probable sites of m^6^A modification. The diagram below the prediction graph is a gene organization map of the entire CVB3 viral RNA. The numbers (1–7) above the graph represent the primer targets used for MeRIP and RT-qPCR, as described in (**B**). (**B**) MeRIP and RT-qPCR were performed on RNAs isolated from CVB3-infected HL-1 cells. The RNAs were sheared into fragments using the AM8740 reagent. MeRIP was carried out using an m^6^A antibody or IgG (control). The precipitated RNAs were then analyzed by RT-qPCR with seven pairs of primers targeting regions of the CVB3 RNA. The graph shows the relative m^6^A levels detected at each target region in comparison to the IgG negative control (data are presented as means ± SEM, n = 3, ns > 0.05, * *p* < 0.05). The locations of the seven qPCR target regions are indicated in Panel (**A**).

To confirm that CVB3 infection induces m^6^A modification of its own genomic RNA and to further map the m^6^A sites on CVB3 RNA, we first performed bioinformatic predictions of the m^6^A sites along the CVB3 genomic RNA using the SRMAP (Qinghua Cui, Peking University, Beijing, China) software [42]. Figure 2A shows that the CVB3 genome has 64 potential m^6^A sites, with more m^6^A sites located near the stop codon and the 3′UTR in particular, and less within the 5′UTR (Supplementary Table S1). This predicted result is in line with the notion that the m^6^A sites on cellular mRNA are mostly located near the stop codon and the 3′UTR [1]. To experimentally confirm and map the distribution of m^6^A sites on CVB3 RNA, MeRIP was conducted to pull down the m^6^A-modified viral RNA fragments using a monoclonal anti-m^6^A antibody (Cell signaling), and RT-qPCR was conducted using seven pairs of specific primers targeting the different regions of the viral genome (Figure 2A and Supplementary Table S2). Figure 2B shows that the levels of m^6^A modifications on CVB3 RNA were significantly increased, particularly at the 3′UTR and the stop codon regions of the CVB3 genomic RNA.

### 3.3. CVB3 Infection Alters Sub-Cellular Localization of m^6^A Modification Machinery

**Figure 3:**
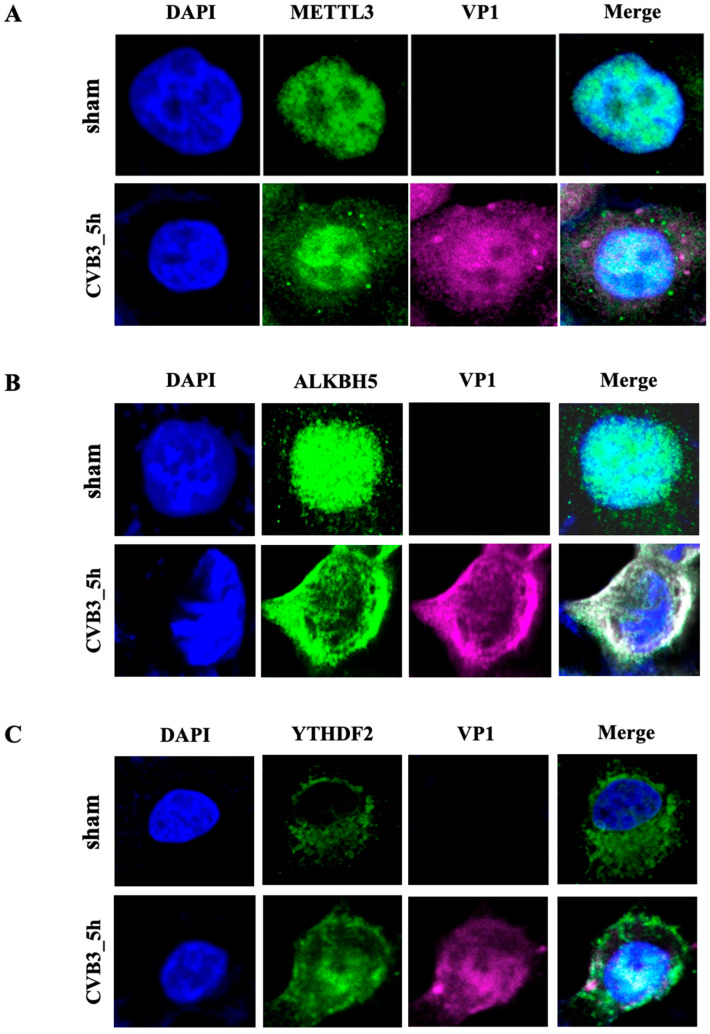
CVB3 infection induces subcellular redistribution of METTL3, ALKBH5 and YTHDF2. (**A**) METTL3 and (**B**) ALKBH5 translocate from the nucleus to the cytoplasm. HeLa cells were infected with CVB3 (10 MOI) or sham-infected with PBS for 5 h. Confocal images show immunofluorescent staining results. The nucleus (blue) was stained with DAPI. Viral protein VP1 (pink) and METTL3 or ALKBH5 (green) were stained using specific antibodies. (**C**) m^6^A reader protein YTHDF2 translocates from the cytoplasm to the nucleus. Cells were treated as described above but stained with an anti-YTHDF2 antibody. Note that at 5 h post infection (hpi), some VP1 also translocated to the nucleus.

Since CVB3 replication occurs in the cytoplasm and previous studies showed that methyltransferases and demethylases co-localize with nuclear speckle markers [12,43,44,45], we next investigated whether CVB3 infection induces the redistribution of m^6^A modification machinery (m^6^A “writer”, “eraser” and “reader” proteins) by immunofluorescent staining and confocal imaging. Figure 3A,B demonstrates that m^6^A methyltransferase METTL3 and demethylase ALKBH5 were mostly concentrated in the nucleus under the sham-infection condition and started to spread to the cytoplasm, co-localizing with CVB3 VP1 protein, at 5 hpi (hours post infection) (note that at 5 hpi, some of the VP1 also moved into the nucleus as VP1 protein contains a nuclear translocation signal sequence at its C-terminus and can be imported into the nucleus at later time points of infection) [46]. However, the reader protein YTHDF2 was located in the cytoplasm under sham-infected conditions and partially migrated to the nucleus at 5 hpi (Figure 3C). All these results suggest that CVB3 infection causes sub-cellular delocalization of the m^6^A modification machinery.

### 3.4. CVB3 Infection Differentially Regulates the Expression of m^6^A Modification Machinery

**Figure 4:**
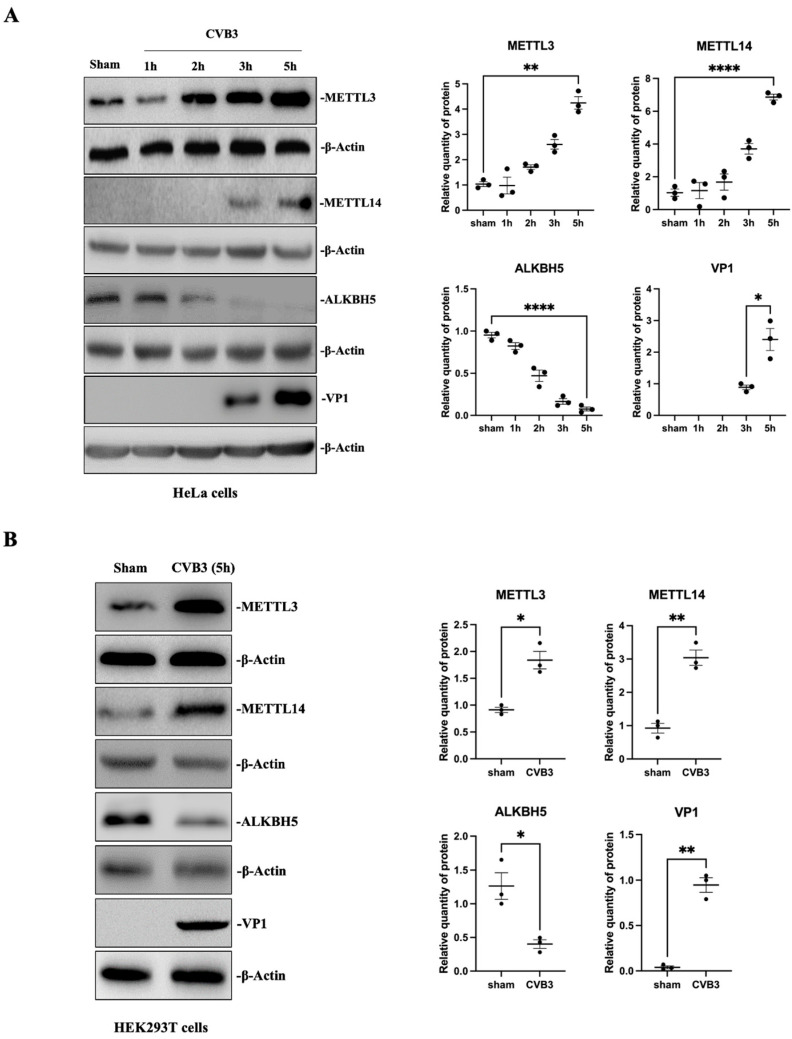
Differential expression of methyltransferases and demethylase in CVB3-infected cells. HeLa cells (**A**) and HEK293T cells (**B**) were infected with CVB3 at an MOI of 10. Cell lysates were collected at the indicated time points pi and analyzed by Western blot using the indicated antibodies. VP1 was used as a marker for CVB3 replication. Quantification of protein expression levels was determined by densitometry using the ImageJ (https://imagej.net/ij/, National Institutes of Health, United States) program. Data were normalized against the β-Actin loading control. Data are presented as means ± SEM, n = 3, * *p* < 0.05, ** *p* < 0.01, **** *p* < 0.0001.

To determine how CVB3 infection regulates the expression of methyltransferase, demethylase and m^6^A reader proteins, we performed Western blot analyses of these protein expression levels in two cell lines infected with CVB3 at 10 MOI or sham-infected with PBS for 5 h. Figure 4A shows that METTL3 and METTL14 were upregulated and ALKBH5 was downregulated at 5 hpi in HeLa cells. Interestingly, the abundance and upregulation of METTL14 expression was much lower than that of METTL3, which supports the notion that METTL3 is a major component of the methyltransferase complex [43,47]. After confirming the time-dependent upregulation of the m^6^A modification machinery, we repeated the experiment in HEK293T cells at 5 hpi. Figure 4B demonstrates a similar expression pattern of these proteins as that in HeLa cells.

### 3.5. METTL3 Expression Increases m^6^A Modification and Promotes CVB3 Replication

**Figure 5:**
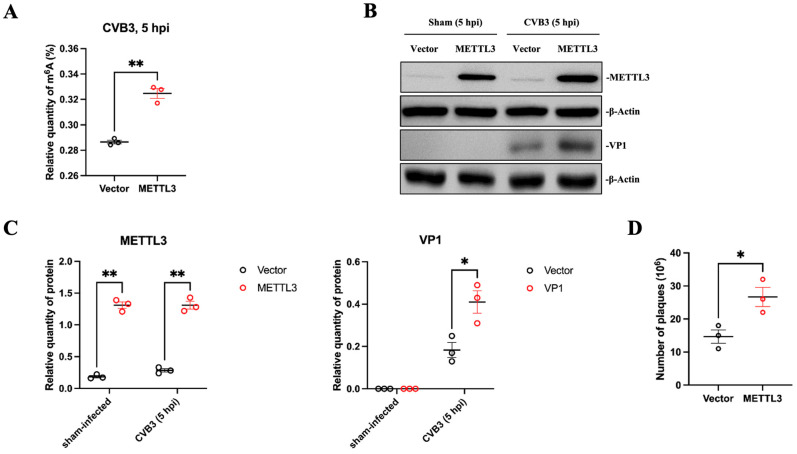

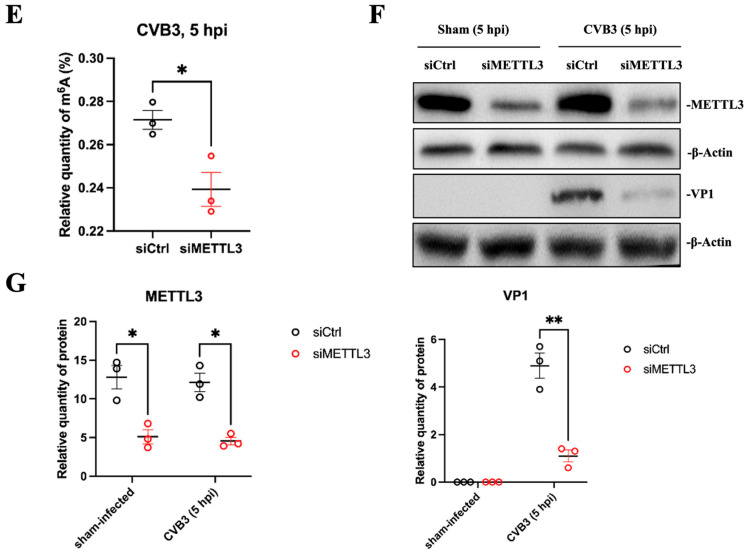
Alteration of METTL3 affects m^6^A modification and VP1 synthesis. (**A**) HeLa cells were transfected with the METTL3 plasmid or an empty vector and then infected with CVB3 or sham-infected with PBS. At 5 hpi, cellular RNAs were isolated for measuring m^6^A levels. (**B**) Cell lysates were used for Western blot analysis of METTL3 and viral VP1 proteins. (**C**) Quantification of protein expression levels was determined by densitometry using the ImageJ program. Data were normalized against the β-Actin loading control. (**D**) Viral plaque assay to detect CVB3 replication using the supernatants from (**B**). (**E**) HeLa cells were treated with METTL3 siRNA (siMETTL3) or scrambled control (siCtrl) siRNA and then infected with CVB3 or sham-infected with PBS. Cellular RNAs were isolated for measuring m^6^A. (**F**) The same cell lysates from (**E**) were used for Western blot analysis of METTL3 and VP1. (**G**) Quantification of protein expression levels was determined as described in (**C**). Data are presented as means ± SEM, n = 3, * *p* < 0.05, ** *p* < 0.01.

As the effect of METTL3-regulated m^6^A modification on viral replication is virus-species-dependent [48,49,50,51], we next determined whether METTL3 expression has an effect on CVB3 replication. To this end, HeLa cells were transfected with a METTL3-expressing plasmid (Addgene) [47] or an empty vector as the control for 36 h and then infected with CVB3 at 10 MOI or sham-infected with PBS for 5 h. Cell lysates were divided into two parts. One part was used for the isolation of cellular RNA as described in Figure 1, and the m^6^A modification levels were measured using the EpiQuik m^6^A Quantification kit (EpiGentek). Figure 5A demonstrates that METTL3 overexpression significantly increased the level of m^6^A methylation. The remaining part of the cell lysates was used to prepare cellular proteins for Western blot analyses of METTL3 and viral protein VP1, a marker of CVB3 replication (Figure 5B,C). The increased VP1 production was further verified by viral plaque assay to measure the produced viral particles using the cell supernatant (Figure 5D). The data indicated that METTL3 overexpression increases m^6^A modification and promotes CVB3 replication.

To further solidify this conclusion, METTL3 expression was silenced by specific siRNAs in HeLa cells. After transfection with METTL3 siRNA or scrambled siRNA for 36 h followed by CVB3 infection at 10 MOI for 5 h, the isolated cellular RNA was used for m^6^A modification analyses (Figure 5E) as described above, and the cellular proteins were prepared for Western blot analysis to detect METTL3 and CVB3 VP1 proteins (Figure 5F,G). The results show that silencing METTL3 significantly reduced m^6^A modification levels and suppressed CVB3 replication, which is evidenced by the reduction in VP1 protein synthesis. These results again suggest that METTL3 plays an important role in catalyzing m^6^A modification and promoting CVB3 replication.

### 3.6. ALKBH5 Expression Decreases m^6^A Modification and Suppresses CVB3 Replication

**Figure 6:**
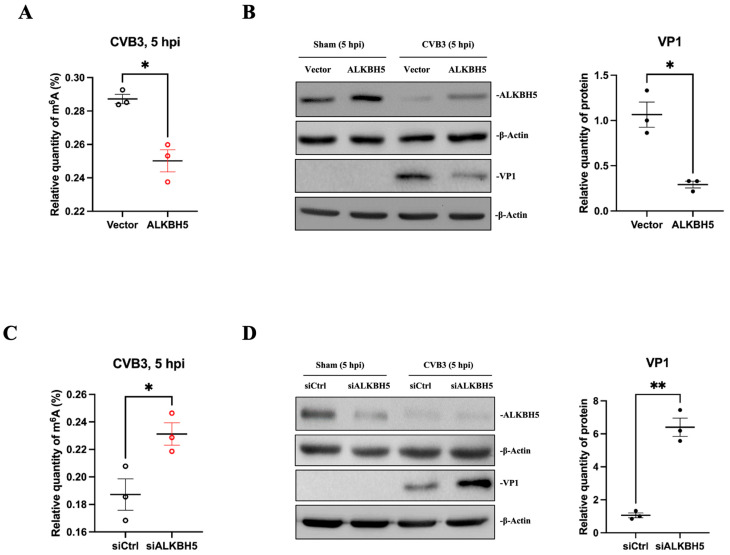
Alteration of ALKBH5 affects m^6^A modification and VP1 synthesis. (**A**) HeLa cells were transfected with the ALKBH5 plasmid or an empty vector and then infected with CVB3 or sham-infected with PBS. Cellular RNAs were isolated for measuring m^6^A levels. (**B**) Cell lysates from (**A**) were used for Western blot analysis of ALKBH5 and viral VP1 proteins. Quantification of protein expression levels was determined by densitometry using the ImageJ program. Data were normalized against the β-Actin loading control. (**C**) HeLa cells were treated with ALKBH5 siRNA (siALKBH5) or scrambled control (siCtrl) siRNA and then infected with CVB3 or sham-infected with PBS. Cellular RNAs were isolated for measuring m^6^A levels. (**D**) The same cell lysates from (**C**) were used for Western blot analysis of ALKBH5 and VP1. Data are presented as means ± SEM, n = 3, * *p* < 0.05, ** *p* < 0.01.

Our next step is to expand our study on the role of the demethylase ALKBH5 in CVB3 RNA m^6^A modification and CVB3 replication by overexpressing or silencing ALKBH5. HeLa cells were transfected with an ALKBH5-expressing plasmid or ALKBH5 siRNA for 36 h and then infected with CVB3 at 10 MOI for 5 h or sham-infected with PBS. The results show that ALKBH5 overexpression decreased the level of m^6^A modification (Figure 6A) and suppressed CVB3 VP1 production (Figure 6B) compared to the vector-only control. However, silencing ALKBH5 with specific siRNAs had the opposite effect (Figure 6C,D), suggesting that ALKBH5 suppresses CVB3 replication.

### 3.7. CVB3 Infection Induces Cleavage of Reader Proteins YTHDF1-3, Benefiting CVB3 Replication

**Figure 7:**
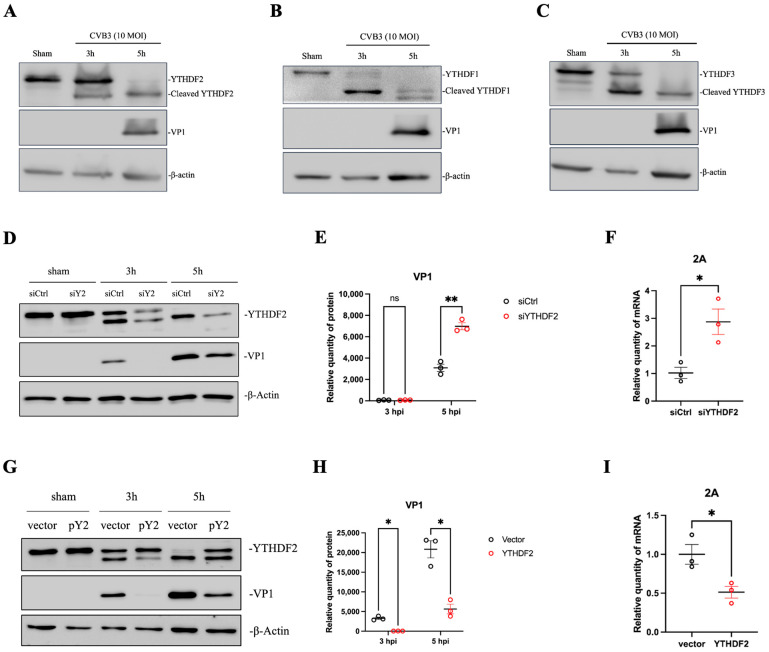
CVB3 infection induces cleavage of reader proteins YTHDF1-3 and increases CVB3 VP1 production and 2A transcription. HeLa cells were infected with CVB3 or sham-infected with PBS. Cell lysates were collected at 3 and 5 hpi for Western blot analysis to detect YTHDF2 (**A**), YTHDF1 (**B**), and YTHDF3 (**C**), as well as viral VP1. (**D**) HeLa cells were transfected with YTHDF2 siRNA (siY2) or control siRNA (siCtrl) and then infected with CVB3. (**G**) HeLa cells were transfected with a YTHDF2 plasmid (pY2) or an empty vector and then infected with CVB3. Western blot was conducted using the indicated antibodies (**D**,**G**), and protein levels were quantified using the ImageJ program (**E**,**H**). RT-qPCR was conducted using samples from (**D**,**G**) to measure the transcripts of the viral gene 2A (**F**,**I**). Data are presented as means ± SEM, n = 3, * *p* < 0.05, ** *p* < 0.01.

m^6^A reader proteins play an important role in RNA metabolism, stability, translation and function [52]. Particularly, YTHDF2, a reader protein that promotes m^6^A RNA degradation [9], is considered an antiviral factor in the setting of viral infection [52]. Our preliminary data indicate a high level of endogenous YTHDF2 expression in uninfected cells. This observation prompted us to investigate the role of YTHDF2 in viral replication and how it is regulated during infection. We hypothesized that YTHDF2 is downregulated, cleaved or degraded upon CVB3 infection. To test this hypothesis, we conducted Western blot analyses using cellular proteins from HeLa cells infected with CVB3 at a MOI of 10 at various time points. The results showed that YTHDF2 is abundant in sham-infected cells but undergoes cleavage in a time-dependent manner, producing fragments of approximately 50 kDa and 20 kDa during CVB3 infection (Figure 7A). In parallel, we assessed the protein levels of YTHDF1 and YTHDF3, finding that both proteins were completely cleaved by 5 hpi (Figure 7B,C).

Viral proteases are known to cleave cellular proteins to manipulate host processes, thereby promoting viral replication. To further explore whether the reduction in YTHDF2 enhances CVB3 replication, HeLa cells were transfected with siRNA to knock down YTHDF2 expression. Following infection with CVB3, we observed a significant increase in viral VP1 levels in siYTHDF2-transfected cells compared to those transfected with scrambled control siRNA (Figure 7D,E). We also found an increase in viral 2A protease gene transcription by RT-qPCR (Figure 7F). Additionally, overexpression of YTHDF2 suppressed VP1 expression and viral RNA transcription, further supporting our conclusion that YTHDF2 plays an inhibitory role in CVB3 replication (Figure 7G–I). In other words, YTHDF2 cleavage during viral infection plays a pivotal role in benefiting viral replication.

### 3.8. Reader Proteins Colocalize and Interact with HuR in SG During the Early Phase of Infection

**Figure 8:**
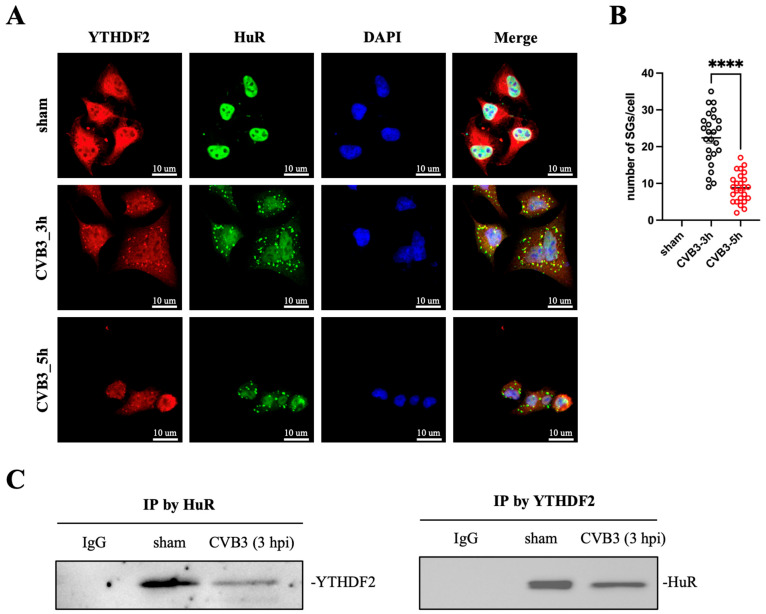
Colocalization and interaction of YTHDF2 with HuR in SGs. (**A**) HeLa cells were either infected with CVB3 (MOI 10) or sham-infected with PBS. The cells were fixed at 3 and 5 hpi and then subjected to immunofluorescent staining for YTHDF2, HuR and DAPI. Images were captured by confocal microscopy. Red indicates YTHDF2, green indicates HuR and blue indicates DAPI. Scale bar: 10 µm. (**B**) The number of SGs per cell was quantified using a total of 25 cells from 5 random microscopic views (n = 25). Data were analyzed by Student’s *t*-test with Welch’s correction and are presented as means ± SEM, **** *p* < 0.0001. (**C**) HeLa cells were infected with CVB3 (MOI 10) for 3 h, and the collected cell lysates were used for co-immunoprecipitation (IP) with HuR antibody. The pulled HuR-protein complexes were detected by Western blot using a YTHDF2 antibody. Conversely, cell lysates were also incubated with the YTHDF2 antibody to pull down the complexes and then detected using the HuR antibody. IgG was used as a control.

To reveal the underlying mechanism by which CVB3-induced RNA m^6^A modification benefits viral replication, we focused on m^6^A reader proteins YTHDF1-3 as it has been recently reported that YTHDF1 and 3 promote SG assembly when exposed to oxidative stress [15]. Since SG is believed to be the platform of host immune response against cellular stress caused by viral infection [23], we hypothesized that reader proteins YTHDF1-3 are involved in the modulation of CVB3 replication via SG formation. To verify this, we first conducted immunofluorescent staining of SG during viral replication to detect the cellular distribution of YTHDF2, using a known SG marker protein HuR [21]. The data revealed that YTHDF2 and HuR co-localized within SGs, with a greater number of SGs observed at 3 hpi compared to 5 hpi (Figure 8A,B). Furthermore, co-immunoprecipitation assay confirmed that YTHDF2 and HuR physically interact with each other (Figure 8C). With these interesting results, we also examined the distribution of YTHDF1 and YTHDF3 within SGs. Similarly, both readers were found to co-localize with HuR (Figure S1).

### 3.9. Silencing m^6^A Machinery Suppresses SG Formation

**Figure 9:**
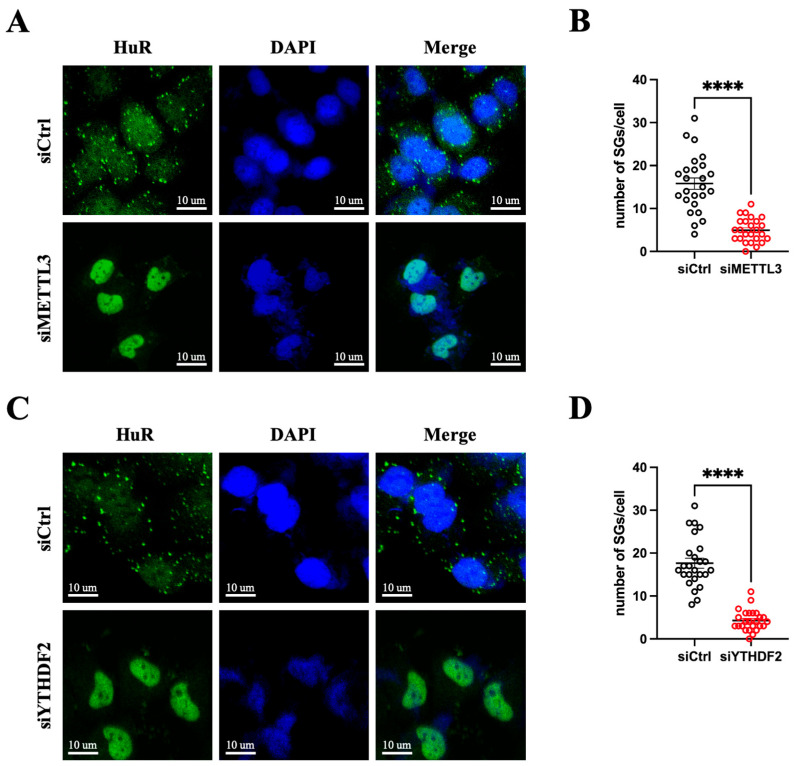
Silencing m^6^A machinery suppresses SG formation. HeLa cells were transfected with specific siRNAs to knock down either METTL3 (**A**) or YTHDF2 (**C**) and then infected with CVB3 at an MOI of 10. Cells were fixed at 3 hpi and then subjected to immunofluorescent staining for HuR and DAPI. Images were captured by confocal microscopy. Green indicates HuR and blue indicates DAPI. Scale bar: 10 µm. (**B**,**D**) The number of SGs per cell was quantified using a total of 25 cells from 5 random microscopic views (n = 25). Data were analyzed by Student’s *t*-test with Welch’s correction and are presented as means ± SEM, **** *p* < 0.0001.

It has been reported that m^6^A-modified mRNAs are preferentially recognized by reader proteins and sequestered in SGs during stress conditions [16]. To investigate whether the m^6^A modification machinery plays a role in CVB3-induced SG formation, we assessed the impact of two key m^6^A machinery proteins, METTL3 and YTHDF2, on this process. HeLa cells were transfected with siRNAs targeting METTL3 or YTHDF2 to silence their expression, followed by infection with CVB3. Surprisingly, immunofluorescent staining showed that knockdown of either METTL3 or YTHDF2 significantly reduced the number of SGs formed during CVB3 infection (Figure 9A–D). These findings suggest that silencing of m^6^A machinery components impairs SG formation, highlighting the linkage between the virus-induced m^6^A modification and the SG formation.

### 3.10. CVB3 3D and dsRNA Are Stored in SG and Colocalize with HuR at the Early Infection Phase

**Figure 10:**
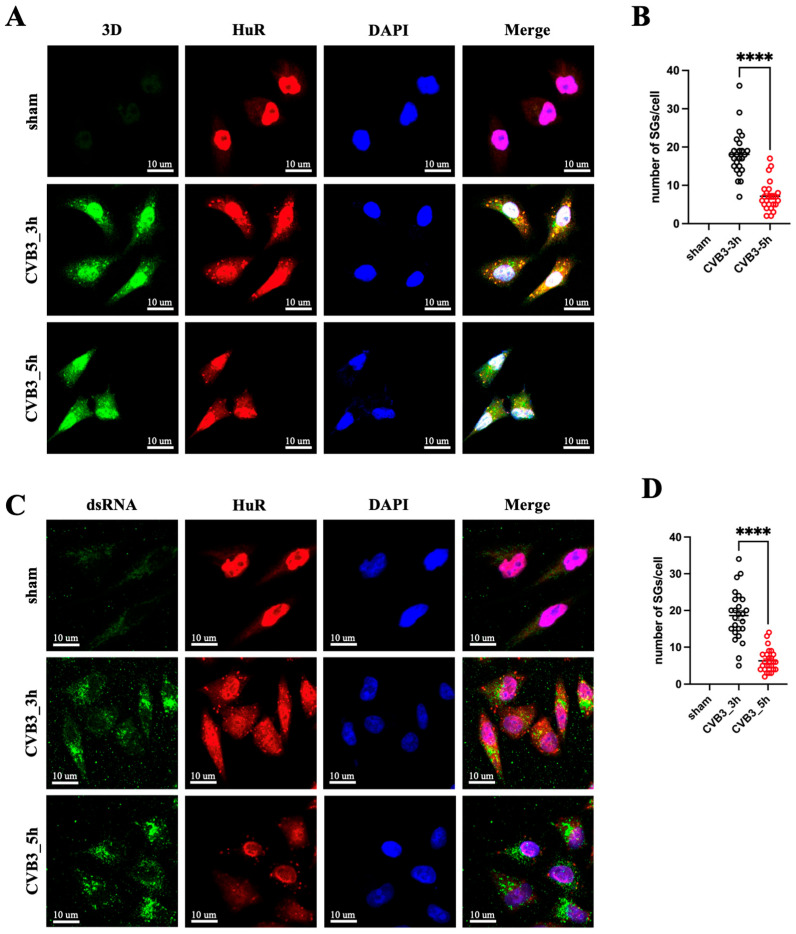
Both CVB3 3D and dsRNA colocalize with HuR in SGs. HeLa cells were either infected with CVB3 (10 MOI) or sham-infected with PBS. The cells were fixed at 3 and 5 hpi and then subjected to immunofluorescent staining for CVB3 3D (**A**), dsRNA (**C**), HuR and DAPI. Images were analyzed by confocal microscopy. Green indicates 3D or dsRNA, red indicates HuR and blue indicates DAPI. Scale bar: 10 µm. (**B**,**D**) The number of SGs per cell was quantified using a total of 25 cells from 5 random microscopic views (n = 25). Data were analyzed by Student’s *t*-test with Welch’s correction. Data are presented as means ± SEM, **** *p* < 0.0001.

CVB3, as a single-stranded RNA virus, replicates in cytoplasm using the RNA-dependent RNA polymerase 3D. To determine whether reader protein-mediated SG assembly affects the cellular distribution of 3D and CVB3 dsRNA, a replicative intermediate of CVB3, during infection, we visualized the dynamic formation of SGs by using HuR, an RNA-binding protein found in SGs [21], as a marker for immunofluorescence staining. The data revealed that 3D (Figure 10A) and CVB3 dsRNA (Figure 10C) co-localize with HuR in SG. The localization of 3D in SG was further confirmed by immunofluorescence staining using G3BP1 antibody, a stress-granule-resident protein (Figure S2). Additionally, we found that CVB3 infection robustly induced SG formation in HeLa cells during the early phase of infection (3 hpi); however, at a later phase (5 hpi), the number of SGs was significantly decreased (Figure 10A,C). The quantification of the SGs in cells is shown in Figure 10B,D.

## 4. Discussion

CVB3 is a cytoplasmic RNA virus that replicates in the cytoplasm using its RNA-dependent RNA polymerase 3D. Recently it has been reported that CVB3 infection induces m^6^A modification and benefits its replication [37]. However, the underlying molecular mechanism is poorly understood. Thus, in this study, we aim to address this issue. By using tissue culture cells and mouse hearts, we first found that CVB3 infection significantly increases m^6^A modification rates of total cellular RNA containing CVB3 RNA. As viral RNA usually contains ~5–7-fold more m^6^A than cellular mRNA [26,47,53], and viral RNA is abundant in host cells after infection, the use of the m^6^A quantification kit EpiGentek to detect m^6^A of CVB3 RNA and total cellular RNA is sensitive and reliable. Importantly, these data were further verified by MeRIP-RT-qPCR using seven pairs of primers specifically targeting different regions of the entire CVB3 genome. Our data show several regions, including the stop codon region, 3′ and 5′ UTR, with peaking levels of m^6^A modification. These results overlap with bioinformatic predictions and are also supported by previous findings on m^6^A site distribution [1,2].

The effect of m^6^A modification on viral replication is viral-species-dependent. m^6^A modifications on viral genomes promote the replication of influenza A virus, SV40, and EV71 [27,50,54] but inhibit the replication of flaviviruses, SARS-CoV2 and the vesicular stomatitis virus [49,51,55]. However, the impact of m^6^A modification on HIV replication is controversial [48,56,57]. Here, we confirmed that m^6^A modifications promote CVB3 replication. This conclusion is based on our data demonstrating that methyltransferase and demethylase expression levels are differentially modulated during CVB3 infection. Specifically, CVB3 infection upregulates METTL3 and METTL14 expression and downregulates ALKBH5. Meanwhile, the expression of CVB3 VP1 protein, a hallmark of CVB3 replication, was significantly increased when methyltransferases were overexpressed, indicating that m^6^A methylation is responsible, at least in part, for the enhanced CVB3 replication. This notion was further solidified by measuring the corresponding m^6^A levels after overexpressing or silencing the methyltransferases or demethylases. In these experiments, the data consistently showed a positive correlation among METTL3 levels, m^6^A methylation rates and CVB3 replication efficiency, while a negative correlation was observed for demethylase ALKBH5. These studies suggest that CVB3-induced m^6^A modifications promote CVB3 replication. In determining the functions of m^6^A writers and erasers during CVB3 replication, we chose METTL3 and ALKBH5 as representatives of the modification machinery based on the following facts: among the three known writer proteins, METTL3, METTL14 and WTAP, METTL3 is the core component of methyltransferase activity and the other two are the activators that strengthen the catalytic effect of METTL3 [47,58]. For the two known eraser proteins, FTO and ALKBH5, both erasers belong to the ALKB family members. However, recent studies suggest that FTO and ALKBH5 have different substrate specificities [12,45]. ALKBH5 removes methyl groups from transcripts containing m^6^A, including viral transcripts [26,50]. However, FTO has a greater substrate specificity, not only acting on mRNAs containing m^6^A, but also on 5′ cap-adjacent nucleotide containing *N6*-methyladenosine, 2′*O*-methylation (m^6^Am) [53,59]. Thus, we only selected ALKBH5 to determine the rate of m^6^A modifications. Other limitations of the study are the small sample size in cell SG analysis and the potential off-target effects in certain siRNA experiments. However, these experiments are all subjected to statistical analyses, and the results are significant.

Reader proteins regulate RNA function by specifically binding to m^6^A-containing motifs on target mRNA. It is widely recognize that reader proteins are the executive factor in modulating the effect of m^6^A modification on various biological processes, such as mRNA metabolism [5,8,9,10], structure switch [11], nuclear export [12] and translation control [13,14]. Recently, reader protein YTHDFs were reported to facilitate SG assembly during cellular oxidative stress conditions [15]. Thus, we investigated the mechanism by which CVB3-induced m^6^A modification enhances viral replication by focusing on the YTHDFs-mediated SG formation. We first found that YTHDF1-3 colocalize and interact with HuR, a known SG marker, at early phases of infection in SGs, but these reader proteins were cleaved at a later phase, resulting in enhanced CVB3 replication (VP1 synthesis and 2A transcription).

In further investigating how CVB3 hijacks host m^6^A modification machinery to benefit its own replication, we demonstrated, for the first time, that both CVB3 RNA polymerase 3D and viral dsRNA are stored in SGs and co-localize with HuR, a key ITAF factor of cellular stress response [31]. It is well-known that the major mechanism involved in stress-activated translation is the IRES-driven initiation and the regulation by ITAFs. HuR, as an ITAF, plays a key role in maintaining the stability of mRNAs and interacts with the target mRNA containing an IRES [60,61]. CVB3, as the positive single-stranded RNA virus, replicates its genome using the 3D polymerase and initiates its RNA translation by an IRES-dependent mechanism [28]. Co-storage of 3D, HuR and CVB3 dsRNA in SGs will potentially protect them from degradation during cellular stress caused by infection. SGs are cytoplasmic aggregates of proteins and mRNA that form in response to various stress conditions to temporarily halt translation and protect mRNAs and other components. The stalled mRNAs and proteins may be reactivated when the stress condition is removed [20,62]. During CVB3 infection, SGs initially serve as a platform of the host antiviral defense by inhibiting viral replication and protein synthesis at early stages of infection. However, CVB3 counteracts this defense by disrupting SG through the cleavage of reader proteins at later phases of infection, allowing the virus to replicate efficiently. Since CVB3 shares a similar genome organization and IRES-driven translation strategy with other enteroviruses, these findings may provide novel insight into the better understanding of the replication mechanism of many other enteroviruses in the *Picornaviridae* family.

In summary, our study provides evidence to show that CVB3 is m^6^A-modified during infection in vitro and in mice. Moreover, we demonstrated that cellular m^6^A modification machinery regulates m^6^A status and plays a positive role in CVB3 replication. Mechanically, we found that CVB3 counteracts host defense through m^6^A modification of its RNA, as well as the cleavage of reader proteins to disrupt SG, leading to the redistribution of the key components (3D, dsRNA and HuR) in cytosol for viral replication. These findings fill a knowledge gap on the regulation of the CVB3 life cycle by m^6^A modifications. Thus, the search for small-molecule inhibitors targeting the m^6^A modification machinery or viral proteases may be a new strategy to develop therapeutic treatments for viral myocarditis.

## Data Availability

All data presented in this study are summarized in the paper. The detailed data of this study are available on a reasonable request from the corresponding author.

## Supplementary Materials

The following supporting information can be downloaded at: https://www.mdpi.com/article/10.3390/microorganisms12112152/s1, Table S1. Prediction of m^6^A sites on CVB3 RNA; Table S2. Primers used to target different regions of CVB3 genome RNA; Figure S1. Both YTHDF1 and YTHDF3 colocalize with HuR in SGs; Figure S2. CVB3 3D colocalizes with G3BP1 in SGs.
