# Supplementary material for: Coxsackievirus B3-Induced m6A Modification of RNA Enhances Viral Replication via Suppression of YTHDF-Mediated Stress Granule Formation
Source: Microorganisms. 2024 Oct 26;12(11):2152. doi: 10.3390/microorganisms12112152 (PMC11596310; doi:10.3390/microorganisms12112152)
Supplement: Supplementary file 1 [file microorganisms-12-02152-s001.zip › microorganisms-3281340-supplementary.pdf]

**Table S1.** Prediction of m$^6$A sites on CVB3 RNA

| # | position | Sequence context | Structural context | Score (binary) | Score (knn) | Score (spectrum) | Score (combined) | Decision |
|---|---|---|---|---|---|---|---|---|
| 1 | 187 | UCAAGCACUUCUGUUACCCCGGA CUGAGUAUCAAUAGACUGCUCA | N/A | 0.675 | 0.664 | 0.533 | 0.618 | M$^6$A site (high confidence) |
| 2 | 202 | ACCCCGGACUGAGUAUCAAUAGA CUG CUCACGCGGUUGAAGGAGA | N/A | 0.663 | 0.671 | 0.572 | 0.627 | M$^6$A site (high confidence) |
| 3 | 484 | AUGCGGCUAAUCCUAACUGCGG ACAGCACACCCUCAAACCAGAGG | N/A | 0.624 | 0.671 | 0.542 | 0.598 | M6A site (moderate confidence) |
| 4 | 770 | AGCUCAAGUAUCAACGCAAAAGA CUGGGGCACAUGAGACCGGGCU | N/A | 0.713 | 0.709 | 0.499 | 0.627 | M6A site (high confidence) |
| 5 | 1101 | GAUAGCGAGGCUACAGCAGAG ACCAACCGACCCAACCAGACGU | N/A | 0.548 | 0.354 | 0.599 | 0.558 | M6A site (moderate confidence) |
| 6 | 1149 | ACAUGUAGGUUCUAUACCCUUGA CUCUGUACAAUGGCAGAAAACC | N/A | 0.544 | 0.740 | 0.630 | 0.588 | M6A site (moderate confidence) |
| 7 | 1212 | AAGCUGCCUGAUGCUUUGUCGA ACUUAGGACUGUUUGGGCAGAAC | N/A | 0.616 | 0.502 | 0.598 | 0.603 | M6A site (high confidence) |

| | | | | | | | | |
|---|---|---|---|---|---|---|---|---|
| 8 | 1219 | CUGAUGCUUUGUCGAACUUAGG ACUGUUUGGGCAGAACAUGCAGU | N/A | 0.694 | 0.608 | 0.612 | 0.657 | M6A site (high confidence) |
| 9 | 1259 | GCAGUACCACUACUUGGGCCGAA CUGGGUAUACCAUACAUGUGCA | N/A | 0.601 | 0.317 | 0.55 | 0.571 | M6A site (moderate confidence) |
| 10 | 1419 | GAUAGCGCCAAAGAGUUUGCGA CAAACCGGUUGCAUCCGGGUCC | N/A | 0.602 | 0.444 | 0.512 | 0.558 | M6A site (moderate confidence) |
| 11 | 1717 | CAAUGUGUGCCGAGUACAAUGG ACUACGUUUGGCCGGGCACCAGG | N/A | 0.707 | 0.644 | 0.608 | 0.664 | M6A site (high confidence) |
| 12 | 1755 | CACCAGGGCUUACCAACCAUGAA CACUCCGGGGAGCUGUCAAUUU | N/A | 0.619 | 0.507 | 0.638 | 0.566 | M6A site (moderate confidence) |
| 13 | 1863 | AGGAUACCUGGUGAGGUGAAGA ACUUGAUGGAAAUAGCUGAGGUU | N/A | 0.645 | 0.714 | 0.706 | 0.673 | M6A site (very high confidence) |
| 14 | 1887 | UUGAUGGAAAUAGCUGAGGUUG ACUCAGUUGUCCCGGUCCAAAAU | N/A | 0.435 | 0.465 | 0.426 | 0.553 | M6A site (low confidence) |
| 15 | 2052 | ACGCUCCUAGGAGAGAUCUUGA ACUAUUACACCCAUUGGUCGGGC | N/A | 0.661 | 0.570 | 0.654 | 0.654 | M6A site (high confidence) |

| | | | | | | | | |
|---|---|---|---|---|---|---|---|---|
| 16 | 2418 | UUCUCUGUCAGGCUAUUGAAGG ACACUCCUUUUAUUUCGCAGCAA | N/A | 0.585 | 0.373 | 0.512 | 0.545 | M6A site (low confidence) |
| 17 | 2442 | ACUCCUUUUAUUUCGCAGCAAA ACUUUUUCCAGGGCCCCGUGGAA | N/A | 0.523 | 0.610 | 0.599 | 0.557 | M6A site (low confidence) |
| 18 | 2499 | GCCGCCAUAGGGAGAGUUGCGG ACACCGUGGGUACAGGGCCAACC | N/A | 0.599 | 0.698 | 0.613 | 0.609 | M6A site (high confidence |
| 19 | 2558 | ACCAGCACUCACUGCUGCUGAG ACAGGUCACACGUCGCAAGUAGU | N/A | 0.450 | 0.616 | 0.648 | 0.538 | M6A site (low confidence) |
| 20 | 2616 | AUGCAGACACGCCACGUUAAGA ACUACCAUUCAAGGUCUGAGUCG | N/A | 0.612 | 0.456 | 0.575 | 0.589 | M6A site (moderate confidence) |
| 21 | 2649 | AGGUCUGAGUCGACCAUAGAGA ACUUCCUAUGUAGGUCAGCAUGC | N/A | 0.575 | 0.483 | 0.599 | 0.580 | M6A site (moderate confidence) |
| 22 | 2960 | UACGAAUCCCAGUGUGUUUUGG ACCGAGGGAAACGCCCCGCCGCG | N/A | 0.572 | 0.618 | 0.548 | 0.562 | M6A site (moderate confidence) |
| 23 | 3133 | AUGUCAAUGCUGGAAGCACGGG ACCAAUAAAAAGCACCAUUAGAA | N/A | 0.543 | 0.635 | 0.592 | 0.567 | M6A site (moderate confidence) |

| | | | | | | | | |
|---|---|---|---|---|---|---|---|---|
| 24 | 3205 | CGUGGAUACCUAGACCACCUAG ACUCUGCCAAUACGAGAAGGCAA | N/A | 0.598 | 0.745 | 0.541 | 0.583 | M6A site (moderate confidence) |
| 25 | 3237 | UACGAGAAGGCAAAGAACGUGA ACUUCCAACCCAGCGGAGUUACC | N/A | 0.625 | 0.712 | 0.529 | 0.591 | M6A site (moderate confidence) |
| 26 | 3307 | UGACAAAUACGGGCGCAUUUGG ACAACAAUCAGGGGCAGUAUACG | N/A | 0.616 | 0.496 | 0.552 | 0.584 | M6A site (moderate confidence) |
| 27 | 3336 | UCAGGGGCAGUAUACGUAGGGA ACUACAGGGUAGUAAAUAGACAU | N/A | 0.571 | 0.633 | 0.555 | 0.568 | M6A site (moderate confidence) |
| 28 | 3529 | ACUACCCAAUUUCAUUUGAAGG ACCAGGUAUAGUAGAGGUCCAAG | N/A | 0.596 | 0.496 | 0.527 | 0.563 | M6A site (moderate confidence) |
| 29 | 3756 | GCAAUGGAACAGGGAGUGAAGG ACUAUGUGGAACAGCUUGGAAAU | N/A | 0.751 | 0.770 | 0.601 | 0.695 | M6A site (very high confidence) |
| 30 | 3852 | AAGGAAUCACUAGUGGGUCAAG ACUCCAUCUUAGAGAAGUCUCUA | N/A | 0.517 | 0.278 | 0.683 | 0.571 | M6A site (moderate confidence) |
| 31 | 4185 | AGGGAAAAACACGAAUUCCUGA ACAGACUCAAACAGCUCCCCCUG | N/A | 0.486 | 0.489 | 0.711 | 0.576 | M6A site (moderate confidence) |

| | | | | | | | | |
|---|---|---|---|---|---|---|---|---|
| 32 | 4189 | AAAAACACGAAUUCCUGAACAG ACUCAAACAGCUCCCCCUGUUAG | N/A | 0.681 | 0.634 | 0.716 | 0.692 | M6A site (very high confidence) |
| 33 | 4931 | CAUCCAAUUCAUAGACAGAAGA ACUCAAGUCAGAUACUCUCUAGA | N/A | 0.6637 | 0.567 | 0.518 | 0.586 | M6A site (moderate confidence) |
| 34 | 5100 | CCACCACCACCACGUAUCGCGG ACUUGCUUAAAUCAGUGGAUAGC | N/A | 0.723 | 0.735 | 0.593 | 0.671 | M6A site (high confidence) |
| 35 | 5403 | GCUGUUGCAAUGAUGAAGAGGA ACUCAAGCACAGUGAAGACAGAG | N/A | 0.606 | 0.609 | 0.562 | 0.589 | M6A site (moderate confidence) |
| 36 | 5494 | UGCCACGUCAUGCCAAACCCGG ACCAACCAUCCUGAUGAAUGACC | N/A | 0.551 | 0.551 | 0.768 | 0.638 | M6A site (high confidence) |
| 37 | 5514 | GGACCAACCAUCCUGAUGAAUGA CCAGGAGGUAGGCGUGCUGGAC | N/A | 0.448 | 0.585 | 0.733 | 0.568 | M6A site (moderate confidence) |
| 38 | 5575 | AUAAGGAUGGUACAAACCUAGA ACUGACACUGCUCAAGUUGAACA | N/A | 0.63 | 0.680 | 0.668 | 0.647 | M6A site (high confidence) |
| 39 | 5579 | GGAUGGUACAAACCUAGAACUG ACACUGCUCAAGUUGAACAGGAA | N/A | 0.485 | 0.605 | 0.667 | 0.564 | M6A site (moderate confidence |

| | | | | | | | | |
|---|---|---|---|---|---|---|---|---|
| 40 | 5595 | GAACUGACACUGCUCAAGUUGA ACAGGAACGAGAAGUUCAGAGAC | N/A | 0.529 | 0.469 | 0.686 | 0.588 | M6A site (moderate confidence |
| 41 | 5616 | AACAGGAACGAGAAGUUCAGAG ACAUCAGAGGCUUCUUAGCAAAG | N/A | 0.576 | 0.566 | 0.681 | 0.617 | M6A site (high confidence) |
| 42 | 5713 | CCAACAUGUACAUUCCGGUGGG ACAAGUCACGGAUUACGGUUUCC | N/A | 0.548 | 0.315 | 0.564 | 0.543 | M6A site (low confidence) |
| 43 | 5978 | UAUUAUCAACACACCUAGUAAG ACUAAGCUGGAGCCGAGUGUCUU | N/A | 0.559 | 0.605 | 0.623 | 0.587 | M6A site (moderate confidence) |
| 44 | 6161 | AGGCUGUUGACCAUUAUGCCGG ACAAUUGGCCACCCUAGACAUUA | N/A | 0.638 | 0.676 | 0.694 | 0.662 | M6A site (high confidence) |
| 45 | 6177 | GCCGGACAAUUGGCCACCCUAG ACAUUAGCACUGAACCAAUGAAG | N/A | 0.522 | 0.694 | 0.723 | 0.611 | M6A site (high confidence) |
| 46 | 6190 | CCACCCUAGACAUUAGCACUGA ACCAAUGAAGUUGGAGGAUGCUG | N/A | 0.382 | 0.614 | 0.749 | 0.540 | M6A site (low confidence) |
| 47 | 6297 | GCCCUGGGCAUCAAGAAGAGAG ACAUCCUCUCAAAGAAGACCAGG | N/A | 0.541 | 0.392 | 0.732 | 0.610 | M6A site (high confidence) |

| | | | | | | | | |
|---|---|---|---|---|---|---|---|---|
| 48 | 6314 | GAGAGACAUCCUCUCAAAGAAG ACCAGGGACCUUACUAAGCUGAA | N/A | 0.416 | 0.471 | 0.718 | 0.519 | M6A site (low confidence) |
| 49 | 6321 | AUCCUCUCAAAGAAGACCAGGG ACCUUACUAAGCUGAAAGAGUGC | N/A | 0.644 | 0.543 | 0.695 | 0.659 | M6A site (very high confidence) |
| 50 | 6394 | UGGUAACCUAUGUGAAAGACGA ACUCAGAUCUGCAGAGAAGGUGG | N/A | 0.654 | 0.616 | 0.781 | 0.783 | M6A site (high confidence) |
| 51 | 6459 | AUUGAGGCGUCCAGUUUGAAUG ACUCUGUGGCAAUGAGACAGACA | N/A | 0.609 | 0.500 | 0.697 | 0.638 | M6A site (high confidence) |
| 52 | 6479 | UGACUCUGUGGCAAUGAGACAG ACAUUCGGCAACUUGUACAAAAC | N/A | 0.546 | 0.372 | 0.660 | 0.582 | M6A site (high confidence) |
| 53 | 6500 | GACAUUCGGCAACUUGUACAAA ACUUUUCACCUAAACCCAGGGGU | N/A | 0.559 | 0.386 | 0.699 | 0.602 | M6A site (moderate confidence) |
| 54 | 6527 | UCACCUAAACCCAGGGGUUGUG ACUGGCAGUGCUGUCGGGUGUGA | N/A | 0.513 | 0.378 | 0.577 | 0.572 | M6A site (low confidence) |
| 55 | 6744 | AACUCCCAUCACCUGUACAGGG ACAAACAUUAUUUUGUGCGGGGU | N/A | 0.622 | 0.451 | 0.519 | 0.572 | M6A site (moderate confidence) |

| | | | | | | | | |
|---|---|---|---|---|---|---|---|---|
| 56 | 6833 | GAUAAAUAACAUCAUAAUCAGG ACACUCAUGCUGAAGGUGUACAA | N/A | 0.638 | 0.756 | 0.614 | 0.634 | M6A site (high confidence) |
| 57 | 6960 | UUGCUUGCUGAAGCUGGCAAGG ACUAUGGAUUAAUCAUGACACCA | N/A | 0.740 | 0.793 | 0.726 | 0.737 | M6A site (very high confidence) |
| 58 | 7019 | GUGCUUCAAUGAAGUUACUUGG ACUAACGUCACAUUCCUAAAGAG | N/A | 0.661 | 0.695 | 0.718 | 0.685 | M6A site (very high confidence) |
| 59 | 7060 | AGAGGUAUUUUAGAGCAGAUGA ACAAUACCCCUUUUUAGUGCACC | N/A | 0.449 | 0.262 | 0.733 | 0.553 | M6A site (low confidence) |
| 60 | 7101 | CACCCCGUUAUGCCCAUGAAAG ACAUACACGAAUCAAUCAGAUGG | N/A | 0.545 | 0.638 | 0.712 | 0.611 | M6A site (high confidence) |
| 61 | 7124 | CAUACACGAAUCAAUCAGAUGG ACCAAGGAUCCAAAGAAUACCCA | N/A | 0.630 | 0.614 | 0.718 | 0.661 | M6A site (high confidence) |
| 62 | 7250 | CGUCCCAGUUGGACGUUGUUUG ACUCUACCUGCGUUCUCAACCAU | N/A | 0.477 | 0.268 | 0.681 | 0.548 | M6A site (low confidence) |
| 63 | 7290 | ACCAUUCGUAGGAAGUGGUUGG ACUCUUUCUAAAUUAGAGACAAU | N/A | 0.654 | 0.291 | 0.595 | 0.612 | M6A site (high confidence) |

| | | | | | | | | |
|---|---|---|---|---|---|---|---|---|
| 64 | 7355 | UUAACCCUACUGUACUAACCGA ACUAGACAACGGUGCAGUAGGGG | N/A | 0.582 | 0.486 | 0.540 | 0.560 | M6A site (moderate confidence) |

**Table S2.** Primers used to target different regions of CVB3 genome RNA

| | | |
|---|---|---|
| Primer 1 | Forward (6637-6654) | Reverse (6758-6774) |
| | CCCTGTCTGGTTTGCTTG | GGCATGCCACCCCTCAC |
| Primer 2 | Forward (6276-6292) | Reverse (6402-6420) |
| | CACTGGGTATCAAGAAG | TTCGCTACCTTCTCTATGG |
| Primer 3 | Forward (3678-3694) | Reverse (3773-3790) |
| | GTGAAGGCGTGGTCGGC | GGAGCCGAATGCATTTCC |
| Primer 4 | Forward (1146-1163) | Reverse (1269-1286) |
| | TTGACTCTGTGCAATGGC | TGCACTGCACATGTACGG |
| Primer 5 | Forward (7039-7055) | Reverse (7129-7146) |
| | GAGGTATTTTAGAGCAG | TGGGTGTTCCTTGGATCC |
| Primer 6 | Forward (7276-7693) | Reverse (7373-7389) |
| | CAGGAAGTGGTTGGACTC | TGCGGAGAATTTACCCC |
| Primer 7 | Forward (1516-1533) | Reverse (1651-1668) |
| | CCAATGGATCAACCTACG | TACGTGGTGGACCCAGGG |

**Figure S1**. Both YTHDF1 and YTHDF3 colocalize with HuR in SGs

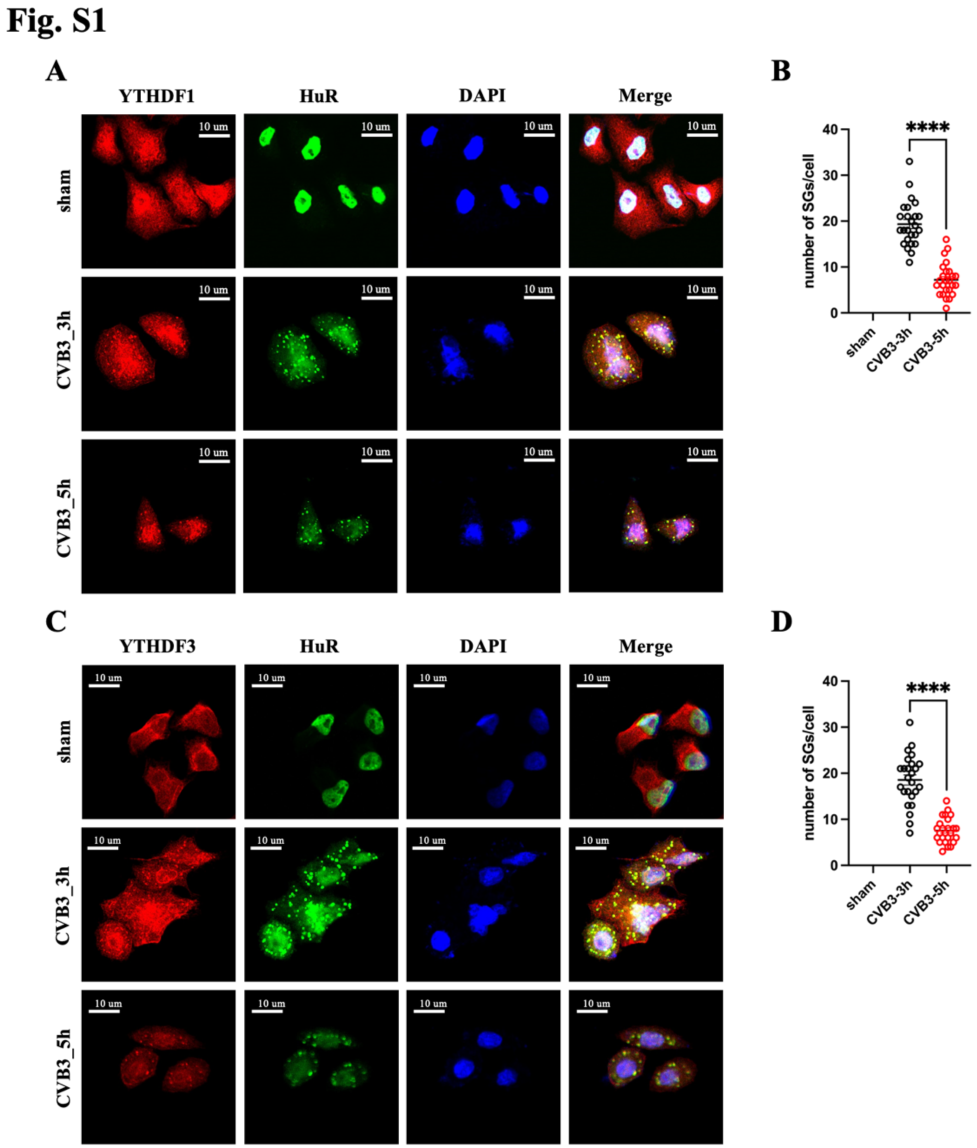

**Fig. S1. Both YTHDF1 and YTHDF3 colocalize with HuR in SGs.** HeLa cells were either infected with CVB3 (MOI 10) or sham-infected with PBS. The cells were fixed at 3 and 5 hpi and then subjected to immunofluorescent staining for YTHDF1 **(A)**, YTHDF3 **(C)**, HuR, and DAPI. Images were captured by confocal microscopy. Scale bar: 10 µm. **(B & D)** The number of SGs per cell was quantified using a total of 25 cells from 5 random microscopic views (n = 25). Data were analyzed by Student's t-test with Welch's correction and are presented as means ± SEM, ****$P$ < 0.0001.

**Figure S2**. CVB3 3D colocalizes with G3BP1 in SGs

## Fig. S2

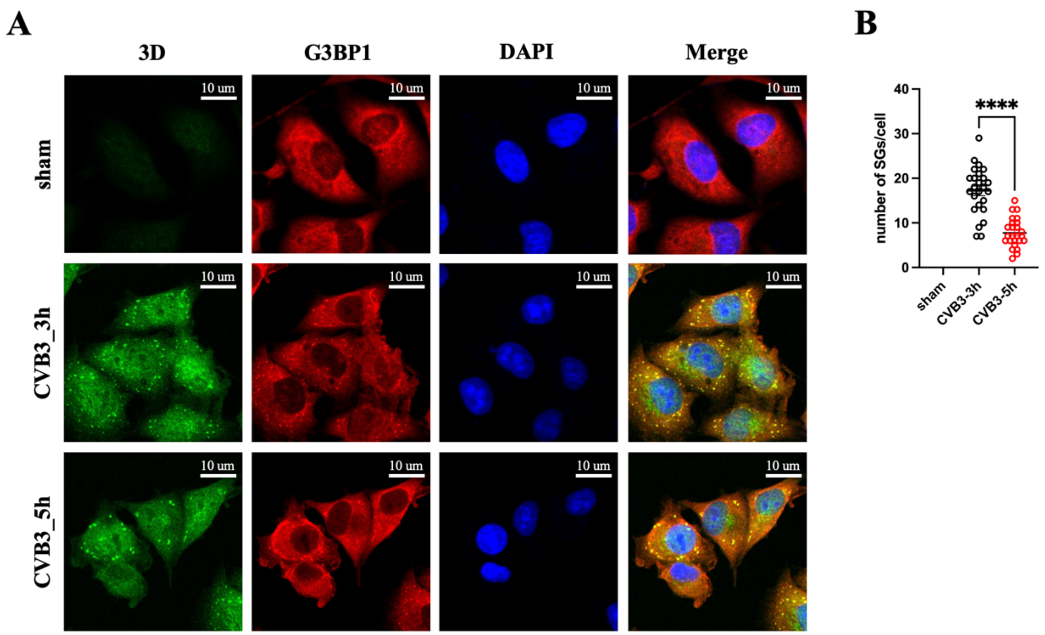


**Fig. S2. CVB3 3D colocalizes with G3BP1 in SGs. (A)** HeLa cells were either infected with CVB3 (10 MOI) or sham-infected with PBS. Infected cells were fixed at 3 and 5 hpi and then subjected to immunofluorescent staining for CVB3 3D, G3BP1, and DAPI. Images were analyzed by confocal microscopy. Scale bar: 10 µm. **(B)** The number of SGs per cell was quantified using a total of 25 cells from 5 random microscopic views (n = 25). Data were analyzed by Student's t-test with Welch's correction and are presented as means ± SEM, ****$P$ < 0.0001.
